# *Gluconacetobacter brunescens* sp. nov., a Novel Acetic Acid Bacterium Isolated from Pear Vinegar, Producing a Water-Soluble Brown Pigment

**DOI:** 10.3390/microorganisms13112620

**Published:** 2025-11-18

**Authors:** Bernarda Karničnik, Igor Jugović, Tomaž Accetto, Lijana Fanedl, Gorazd Avguštin, Janja Trček

**Affiliations:** 1 Department of Biology, Faculty of Natural Sciences and Mathematics, University of Maribor, 2000 Maribor, Slovenia; bernarda.karnicnik1@gmail.com (B.K.); igorjugovic444@gmail.com (I.J.); 2 Department of Microbiology, Biotechnical Faculty, University of Ljubljana, 1000 Ljubljana, Slovenia; tomaz.accetto@bf.uni-lj.si (T.A.); lijana.fanedl@bf.uni-lj.si (L.F.); gorazd.avgustin@bf.uni-lj.si (G.A.)

**Keywords:** acetic acid bacteria, *Acetobacteraceae*, *Gluconacetobacter*, *Gluconacetobacter brunescens*, pear vinegar, natural pigments, melanin

## Abstract

The clade *Gluconacetobacter* comprises eleven species originating from various sources such as rhizosphere soil, pink sugarcane mealybug, and vinegar. During sampling of organic vinegars, we isolated strain Hr-1-5, which exhibits high 16S rRNA gene sequence identities (≤98.6%) and low 16S-23S rRNA gene internal transcribed spacer (ITS) sequence identities (≤92.1%) with *Gluconacetobacter* species. Further genome analysis confirmed that strain Hr-1-5 is a distinct species, supported by an average nucleotide identity (ANIb) of ≤90.6% and an in silico DNA–DNA hybridization (dDDH) value of ≤46% compared with other recognized *Gluconacetobacter* species. Strain Hr-1-5 darkens the growth medium to a deep brown after 4–5 days of submerged cultivation and similarly colors agar medium after 5–6 days. In silico genome analysis suggests that the strain synthesizes pyomelanin. Phenotypically, it is distinguished from its closest *Gluconacetobacter* relatives by its ability to produce 5-keto-D-gluconic acid, but not 2-keto-D-gluconic acid, and by its capacity to grow on D-ribose, among other traits. These findings support the classification of strain Hr-1-5 as a novel species, for which we propose the name *Gluconacetobacter brunescens* sp. nov. Hr-1-5^T^ (=ZIM B1168^T^ = LMG 33629^T^). Strain Hr-1-5 is of biotechnological interest for its pigment production and enables the in situ production of colored cellulose in a co-culture with a cellulose-producing acetic acid bacterium.

## 1. Introduction

The genus *Gluconacetobacter* is a member of the family *Acetobacteraceae* within the class *Alphaproteobacteria* [1]. It currently comprises twelve validly published species, with *Gluconacetobacter liquefaciens* designated as the type species of the genus [2]. Although *Gluconacetobacter entanii* is phylogenetically affiliated with the genus *Novacetimonas*, it has retained its original taxonomic designation due to the inability to revive the type strain [3,4].

Members of *Gluconacetobacter* are acetic acid bacteria (AAB), a group that has undergone extensive taxonomic revision in recent decades [5]. Species characterized by ubiquinone 10 (UQ-10) as the dominant respiratory quinone were originally placed in the genus *Acetobacter* but were subsequently transferred to *Gluconacetobacter* [6]. Phylogenetic analyses have revealed two principal clades within the genus: one represented by *G. liquefaciens* and another by *Gluconacetobacter xylinus*. In 2012, the genus *Komagataeibacter* was established, and the clade containing *G. xylinus* and its relatives was reclassified accordingly [7].

Species of the genus *Gluconacetobacter* have been isolated from a wide range of environments, including fruit [8], rhizosphere soil [9], the interior of stone chamber [10], the leaf sheath of sugarcane, and the pink sugarcane mealybug [11]. Members of this genus exhibit promising potential in various biotechnological applications, such as agriculture [12,13], bacterial cellulose production [14], and as a component of dietary supplements with potential as antibiotic alternatives in aquaculture, specifically of olive flounder (*Paralichthys olivaceus*) [15].

Pigment-producing bacteria have attracted substantial industrial and biomedical interest due to their natural origin and their demonstrated antioxidant and antibacterial activities [16]. Among these, *Gluconacetobacter diazotrophicus* was identified as the first known bacterial producer of betalains, pigments previously considered to be restricted to the plants and certain fungi [17]. This discovery significantly broadened the known phylogenetic distribution of betalain biosynthesis. Betalains are hydrophilic nitrogen-containing pigments that are subdivided into two subclasses: yellow–orange betaxanthins, formed through condensation with amines or amino acids, and the red–violet betacyanins, derived from conjugation with (2S)-5,6-dihydroxy-2-indolinecarboxylic acid (cyclo-DOPA). These pigments are valued for their intense colors and strong antioxidant properties. In addition, they have also been shown to suppress the proliferation of various cancer cell lines in a dose-dependent manner and to exhibit broad-spectrum antimicrobial activity [18].

Central to the biosynthetic pathway of betalains is the enzyme 4,5-L-DOPA-extradiol-dioxygenase (DODA), which catalyzes the ring cleavage of L-3,4-dihydroxyphenylalanine (L-DOPA) to yield betalamic acid, the chromophore core and universal precursor of all betalains pigments. This dioxygenase was first biochemically characterized in the cyanobacterium *Anabaena cylindrica* and subsequently identified in *G. diazotrophicus*. The bacterial DODA from *G. diazotrophicus* (GdDODA) exhibits both 2,3- and 4,5-ring-cleavage activities and demonstrates superior catalytic efficiency relative to plant DODA homologs, including those from *Beta vulgaris* (e.g., lower K_m_, higher V_max_). Heterologous expression of GdDODA in microbial hosts or transient expression in plant tissues such as *Nicotiana benthamiana* efficiently produces betalamic acid and betalains, supporting the notion for convergent evolution of betalain-forming dioxygenases across kingdoms [18]. Owing to their vivid coloration and bioactivity, these pigments hold considerable promise as natural food colorants and as functional ingredients in pharmaceutical formulations and dietary supplements [17,19].

Melanins are another class of pigments, dark-colored polymers with multifunctional roles in protection, including defense against ultraviolet radiation, reactive oxidative species, and toxic metal ions, and they have also been implicated in microbial virulence [20]. Among the recognized subclasses, eumelanin represents the classic brown-to-black pigment formed via oxidative polymerization of the amino acids tyrosine and/or phenylalanine through the precursor L-DOPA, which is subsequently converted to dopachrome and then to the mature melanin polymer [21]. In *Gluconacetobacter tumulisoli* FBFS 97, researchers isolated a brown pigment whose structural characteristics, detection of L-DOPA intermediates, and genome analyses suggested it is eumelanin. Targeted disruption of specific biosynthetic genes (e.g., *pheA*) increased pigment yield, indicating a genetically encoded pathway for eumelanin synthesis in this AAB [22]. Beyond eumelanin, other structural variants include pheomelanin and pyomelanin. Pheomelanin is a sulfur-containing, red or yellow pigment produced through a pathway analogous to that of eumelanin but involving cysteinylation of L-DOPA intermediates, which imparts its characteristic reddish coloration [21]. Pyomelanin, by contrast, is a nitrogen-free, water-soluble brown melanin-like polymer derived from homogentisic acid, a downstream catabolite of tyrosine metabolism, and is classified within the broader allomelanin group [21,23].

In this study, we present a polyphasic taxonomic analysis that supports the delineation of strain Hr-1-5 as a novel species within the genus *Gluconacetobacter*. We propose the name *Gluconacetobacter brunescens* for this species, with strain Hr-1-5 designated as the type strain. Strain Hr-1-5 exhibits the ability to produce a dark brown pigment that is readily secreted into the growth media. Whole genome sequencing and in silico metabolic reconstruction revealed putative biosynthetic pathways for this pigment. Furthermore, co-culture experiments with a bacterial cellulose (BC) producing AAB demonstrated in situ formation of colored BC, highlighting the biotechnological potential of this newly characterized species.

## 2. Materials and Methods

### 2.1. Isolation of Hr-1-5 and Growth Conditions

Strain Hr-1-5 was isolated from organic pear vinegar sampled at a farm in the Pohorje region of Northeast Slovenia (46°31′38.439″ N 15°27′47.873″ E). Isolation was performed on RAE medium containing glucose (40 g/L), peptone (10 g/L), yeast extract (10 g/L), citric acid (1.37 g/L), Na_2_HPO_4_ × 2H_2_O (3.38 g/L), 1% (*v*/*v*) ethanol and 1% (*v*/*v*) acetic acid, during a survey of organic vinegars [24]. The isolate was routinely incubated at 30 °C for 48 h under high relative humidity. For long-term preservation, strain Hr-1-5 was stored in RAE liquid medium supplemented with 20% (*v*/*v*) glycerol at −80 °C.

### 2.2. Molecular Identification of Hr-1-5

Strain Hr-1-5 was cultured on RAE medium supplemented with 1% (*v*/*v*) acetic acid and 1% (*v*/*v*) ethanol. After 2 days of incubation, biomass was harvested, and genomic DNA was extracted using PrepMan^TM^ reagent (Thermo Fisher Scientific, Waltham, MA, USA), following the manufacturer’s protocol. The 16S rRNA gene sequence was PCR amplified with the primers 27f (5-AGAGTTTGATCMTGGCTCAG-3) and rH1542 (5-AAGGAGGTGATCCAGCCGCA-3). The cycling program started with initial denaturation of DNA at 95 °C for 3 min and continued with 30 cycles of 95 °C for 30 s, 58 °C for 30 s and 72 °C for 1 min. Additionally, the internal transcribed spacer (ITS) region between the 16S and 23S rRNA genes was amplified using the primer pair SpaFw (5′-TGCGGCTGGATCACCTC-3′) and SpaRev (5′-GTGCCAAGGCATCCACC-3′) as previously described [25]. PCR products were purified using the NucleoSpin Gel and PCR Clean-up Mini kit (Macherey Nagel, Düren, Germany). Sanger sequencing was performed by Microsynth (Vienna, Austria), and the resulting sequences were analyzed using the BLAST tool (v2.15.0) available on the NCBI database.

### 2.3. Genome Analysis

Biomass was submitted to MicrobesNG (Birmingham, UK) for hybrid genome sequencing using both Illumina and Oxford Nanopore platforms. Genomic DNA libraries for Illumina sequencing were prepared using the Nextera XT Library Prep Kit (Illumina, San Diego, CA, USA), according to the manufacturer’s instructions. DNA quantification and library construction were performed using a Hamilton Microlab STAR automated liquid handling system (Hamilton Bonaduz AG, Bonaduz, Switzerland). Sequencing was carried out on the Illumina NovaSeq 6000 system (Illumina, San Diego, CA, USA) employing a 250 bp paired-end read configuration. Adapter trimming was performed using Trimmomatic version 0.30. For long-read sequencing, high-molecular-weight DNA (200–400 ng) was used to prepare libraries with the SQK-RBK114.96 kit from Oxford Nanopore Technologies (ONT, Oxford, UK). Barcoded samples were pooled into a single library and sequenced on a FLO-MIN114 (R10.4.1) flow cell using a GridION platform (ONT, Oxford, UK). Hybrid genome assembly was performed using Unicycler version 0.4.0 [26], and genome annotation was performed using Prokka version 1.11 [27]. Average nucleotide identity (ANI) values were calculated using the JSpecies software tool (v5.0.2) [28]. Genome-to-genome distances between strain Hr-1-5 and publicly available reference genomes were estimated using the Genome-to-Genome Distance Calculator (GGDC) 3.0 [29]. Prophage regions were predicted using Cenote-Taker2 (v2.1.5) [30] and PHASTEST (v3.0) [31], as using multiple tools is more reliable [32]. A phylogenetic tree was constructed based on core genome sequences using the maximum likelihood method PhyML (v3.3.20250515) [33], employing the GTR nucleotide substitution model and 1000 bootstrap replicates.

### 2.4. Phenotypic Analysis

Phenotypic characterization was performed as previously described by Škraban et al. [34]. Shortly, strains were cultivated aerobically at 30 °C on RAE medium with 1% (*v*/*v*) ethanol and 1% (*v*/*v*) acetic acid. Catalase and oxidase activities were assessed by standard methods. Carbon source utilization (D-ribose, sorbitol, D-mannitol, glycerol, D-gluconate, 1-propanol, ethanol) was assessed on solid medium (1% selected carbon source, 0.5% yeast extract, 1.5% agar, pH 6.8). Growth in 30% glucose was monitored over two weeks, in media composed of 0.5% yeast extract, 30% glucose and 1.5% agarose. The assimilation of selected sources with ammonium sulfate as the sole nitrogen source was tested on Hoyer–Frateur (0.1% (NH_4_)_2_SO_4_, 0.09% KH_2_PO_4_, 0.01% K_2_HPO_4_, 0.025% MgSO_4_ × 7H_2_O, 0.002% FeCl_3_ × 6H_2_O, 3% of carbon source, pH 6.8) and Asai (0.3% (NH_4_)_2_SO_4_, 0.3% KH_2_PO_4_, 0.2% MgSO_4_ × 7H_2_O, 5% of carbon source, pH = 6.8) media. Tolerance to ethanol and acetic acid was assessed in liquid RAE medium [34].

Gluconic acid identification followed the protocol of Marič et al. [35]. Briefly, the strains were initially grown on GY (5% glucose and 0.5% yeast extract) agar for 5 days. Afterward, a single colony was transferred to a liquid medium containing 2% glucose and 2% sodium gluconate and incubated at 30 °C with shaking (180 rpm) for 11 days. Keto-gluconic acids were separated using silica gel 60 TLC plates (Merck Millipore, Burlington, MA, USA) with a mobile phase of ethyl acetate, acetic acid, methanol, and water (6:1.5:1.5:1). The acids were visualized using a 2% diphenylamine suspension.

Fatty acid composition was analyzed from biomass grown on RAE medium supplemented with 1% (*v*/*v*) ethanol and 1% (*v*/*v*) acetic acid, following the procedure described by Marič et al. [35]. Briefly, cultivation was carried out for 48 h at 30 °C under aerobic conditions. Cell inoculation, harvesting, extraction, and analysis of fatty acid methyl esters followed the standard protocol of the MIDI system (Sherlock Microbial Identification System, Inc., Newark, DE, USA). The esters were separated by gas chromatography (Agilent 6890, Santa Clara, CA, USA) and identified using the aerobe database RTSBA6 (Sherlock v. 6.1).

The ability of strain Hr-1-5 to grow in the presence of 10% ethanol, as well as in media containing 1% and 2% NaCl, was evaluated using the method of Sombolestani et al., by using the standard SM medium (0.5% yeast extract and 5% D-glucose) [8]. Growth of strain Hr-1-5 on media lacking ethanol and acetic acid was evaluated on mannitol agar (MA; 25 g/L mannitol, 5 g/L yeast extract, 3 g/L peptone and 15 g/L agar), glucose yeast agar (GY; 50 g/L glucose, 5 g/L yeast extract and 15 g/L agar), and RAE-agar (without acetic acid and ethanol) after 48 h of incubation inoculated plates at 30 °C. The strain’s tolerance to varying concentrations of acetic acid and ethanol in liquid RAE medium was tested in test tubes against 0.5% ethanol with 0.5%, 1%, 1.5%, 2%, 2.5%, and 3% acetic acid, and RAE medium containing 1% ethanol with 0.5%, 1%, 1.5%, 2.5% and 3% acetic acid. Cultures were incubated at 30 °C and 180 rpm for seven days. Growth was considered positive if the optical density at 600 nm exceeded 0.2, weak if the optical density ranged from 0.07 to 0.2, and absent if it was below 0.07.

The ability to grow under microaerobic and anaerobic conditions was evaluated using commercial atmosphere generators GENbox microaer and GENbox anaer (bioMérieux, Marcy-l′Étoile, France) following the manufacturer’s protocol. The bacteria were inoculated on RAE agar supplemented with 1% (*v*/*v*) ethanol and 1% (*v*/*v*) acetic acid and incubated. Growth was evaluated after 5 days of incubation at 30 °C. The assay was repeated three times [24]. Growth was defined as strong if colonies were too numerous to count, and weak if colony numbers were below 300 [24].

Bacterial motility was assessed on semi-solid RAE agar plates containing 0.8% (*v*/*v*) agar. A single colony from a 3-day-old culture on RAE agar was inoculated by gently stabbing the center of the plate with a sterile needle. Plates were then incubated at 30 °C for 5 days. Motility was evaluated as described by Tittsler and Sandiholzer [36]. Shortly, the assay was based on radial colony expansion from the inoculation point and classified as motile if the spread exceeded the stab line, causing turbidity throughout the medium in a diffuse or cloud-like pattern. Non-motile bacteria exhibited growth only along the line of inoculation without spreading into the surrounding medium.

The production of bacterial nanocellulose was assessed as described previously [37]. Briefly, pellicle formation was initiated by inoculating a single colony into 50 mL of RAE medium in 250 mL baffled Erlenmeyer flasks sealed with 0.2 µm membrane screw caps. Cultures were shaken at 180 rpm for 24 h at 30 °C, followed by static incubation for 3 days at 30 °C.

Antimicrobial resistance was assessed using the disk diffusion method, adapted from EUCAST guidelines, as previously described by Cepec and Trček [38]. Briefly, strains were precultured for 3 days at 30 °C on RAE medium with 1% (*v*/*v*) ethanol and 1% (*v*/*v*) acetic acid under high humidity. Biomass was suspended in 0.85% NaCl and adjusted to 0.5 McFarland. The suspension was evenly spread onto agar plates (RAE with 1% ethanol and 1% acetic acid) using sterile cotton swabs. Antibiotic disks (BioRad, Hercules, CA, USA) applied were: gentamicin, ampicillin, chloramphenicol, ciprofloxacin, erythromycin, and trimethoprim. Inhibition zone diameters were measured after 2 days of incubation at 30 °C under high humidity [38].

To evaluate the potential inhibitory effect of strain Hr-1-5 on pathogenic bacteria, co-cultivation assays were performed with *Escherichia coli* ATCC 25922, *Enterococcus faecalis* ATCC 29212, *Staphylococcus aureus* ATCC 29213, and *Komagataeibacter melomenusus* AV436^T^. Strains Hr-1-5 and *G. tumulicola* LMG 27725^T^ were streaked on one side of an RAE medium plate (without acetic acid and ethanol) and incubated at 30 °C for five days. During incubation, a brown pigment was observed diffusing into the medium. After this period, the test strains were inoculated on the opposite side of the same plates, and their growth was monitored at 24, 48, and 72 h post-inoculation.

### 2.5. Metabolic Pathway Analysis

The metabolic pathway was reconstructed by integrating data from the KEGG database [39] and Reactome pathway database [40]. The pathway was defined with the biosynthesis of L-tyrosine as the starting point and the pigment molecules or their degradation products as the terminal nodes. L-DOPA was placed at the center of the network, and the initial reactions were represented manually, forming a circular core involving three key intermediates: L-DOPA, DOPA quinone, and cyclo-DOPA [41,42]. From this central triad, the pathway was extended both upstream toward L-tyrosine and tyrosinase, and downstream toward the final pigment products. An additional branch for pyomelanin biosynthesis, not represented in either KEGG or Reactome, was incorporated based on a recent study [22].

To predict the metabolic path of the brown pigment produced by strain Hr-1-5, we combined pathway reconstruction with sequence-based evidence. Protein sequences of enzymes annotated in the relevant pathways were retrieved from Hr-1-5 (GenBank accession numbers WP_420358039.1, WP_420358206.1, WP_420358592.1, WP_420357148.1, WP_420355655.1, WP_420357753.1, WP_420355788.1, and WP_420356861.1). Functional annotations (enzyme names and EC numbers) guided their selection. These sequences were subjected to BLASTp analysis, and results were evaluated based on query coverage, E-value, and percent identity. Comparative analyses included bacterial representatives from the genera *Novacetimonas*, *Komagataeibacter*, *Gluconacetobacter*, *Streptomyces*, *Pseudomonas*, *Bacillus*, *Acinetobacter*, and the species *Escherichia coli*. Plant representatives were also included, specifically species from the genera *Amaranthus* and *Bougainvillea*, and *Silene latifolia*, *Spinacia oleracea*, *Beta vulgaris*, *Mirabilis jalapa*, and *Portulaca grandiflora.* From the 8 bacterial groups, we selected the following representatives:

(a) *Novacetimonas hansenii* (No. 1), *Komagataeibacter xylinus* (No. 2) and *Escherichia coli* for negative control, displaying no natural pigment production;
(b) *Gluconacetobacter brunescens* (No. 3), *Gluconacetobacter tumulicola* (No. 4) and *Gluconacetobacter liquefaciens* (No. 7) for water-soluble brown pigment production [10,43];
(c) *Gluconacetobacter tumulisoli* (No. 5) for eumelanin production [22];
(d) *Gluconacetobacter diazotrophicus* (No. 6) for betalain production [17];
(e) *Streptomyces scabiei* (No. 8) for multiple melanin production [44];
(f) *Pseudomonas putida* (No. 9) and *Pseudomonas aeruginosa* (No. 10) for pyomelanin production and production of other pigments, such as pyocyanin, pyoverdine and pyorubin [16,45,46,47];
(g) *Acinetobacter baumannii* (No. 11) just for pyomelanin production [48].

The six plant groups were chosen because of their known ability of betalain production or taxonomically close relationship to *Caryophyllales* [49,50,51,52,53,54,55,56,57,58].

For an aligned sequence to be considered functionally equivalent, its annotation had to match that of the query sequence. Similarity thresholds (E-value, percent identity) were not used as primary criteria, even when statistical scores were suboptimal.

The BLAST results were organized into a dataset. The first column listed the independent variable (species), represented by 10 samples across the groups described above. The subsequent 24 columns contained BLAST parameters: query coverage (Cov), E-value (E), and percent identity (ID) for each of the eight enzymes (P1 = WP_420358039.1, P2 = WP_420358206.1, P3 = WP_420358592.1, P4 = WP_420357148.1, P5 = WP_420355655.1, P6 = WP_420357753.1, P7 = WP_420355788.1, P8 = WP_420356861.1). For *G. brunescens* Hr-1-5, all enzymes showed 100% query coverage, E-value of 0.0, and 100% identity. In cases where an enzyme was absent or represented only marginally, values were assigned as 0% coverage, 1.0 E-value, and 0% identity. To analyze these data, we developed a custom Python script, named MetaPathClust (short for Metabolic Pathway Clustering), designed for dataset processing, enzyme sequence clustering, and pathway comparison. The workflow, available at https://github.com/i-jugovic/MetaPathClust.git (accessed on 15 August 2025), supports multiple applications: comparative enzymology (cross-species metabolic similarity analysis), phylogenetic analysis (contrasting taxonomic versus functional clustering), biomarker discovery (identification of enzyme signatures distinguishing taxonomic groups), evolutionary studies (patterns of enzyme conservation) and synthetic biology (selection of candidate enzymes for pathway engineering).

### 2.6. Production of Colored BC Membranes

*Gluconacetobacter brunescens* Hr-1-5, a pigment-producing strain, and *Komagataeibacter melomenusus* AV436^T^, a bacterial nanocellulose-producing strain [35], were used for cocultivation experiments. A single large colony of each strain was inoculated into 50 mL of RAE medium supplemented with 1% (*v*/*v*) ethanol and 1% (*v*/*v*) acetic acid, in a 250 mL baffled Erlenmeyer flask with three-hole-membrane (0.2 μm) screw caps (DWK Life Sciences, Mainz, Germany). The cultures were incubated under aerobic conditions at 30 °C with shaking (180 rpm) for 24 h, followed by static incubation for 14 days.

## 3. Results and Discussion

### 3.1. Morphological, Physiological and Biochemical Tests

**Figure 1:**
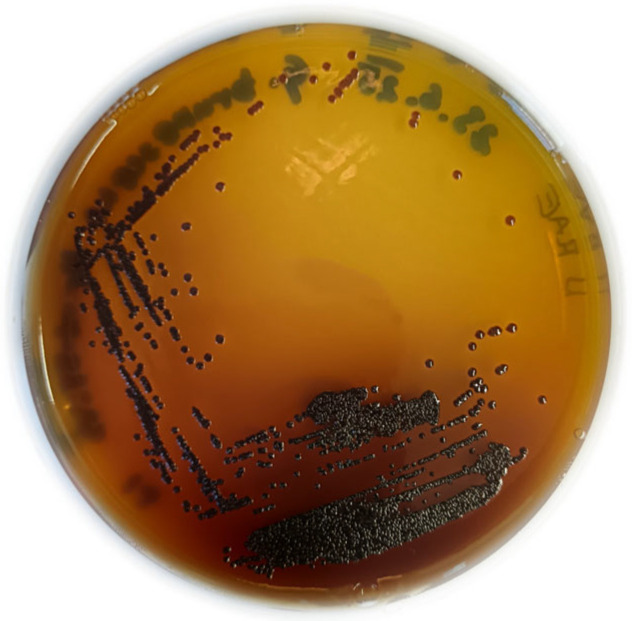
*Gluconacetobacter brunescens* Hr-1-5^T^ after 5–6 days of growth on RAE agar supplemented with 1% ethanol and 1% acetic acid.

**Figure 2:**
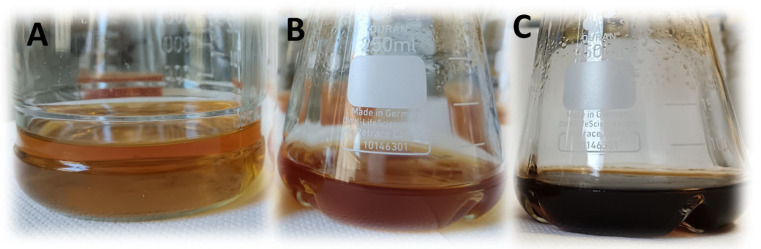
*Gluconacetobacter brunescens* Hr-1-5^T^ after 7 days (**B**) and 14 days (**C**) of growth in RAE medium supplemented with 1% ethanol and 1% acetic acid. For reference, a flask containing uninoculated RAE medium is shown (**A**).

Standard microbiological analysis revealed that strain Hr-1-5 is a Gram-negative bacterium, catalase-positive, and oxidase-negative, which are traits characteristic of AAB [5]. After 5–6 days of incubation on RAE agar supplemented with 1% ethanol and 1% acetic acid under aerobic conditions at 30 °C, colonies reached approximately 0.4 mm in diameter. The colonies were round, brown in color, raised, and had smooth edges (Figure 1). After one week of incubation the colonies develop a dark brown coloration, accompanied by pigment extrusion into the surrounding growth medium, which through the course of another week darkens even more (Figure 2).

Strain Hr-1-5 exhibited mild tolerance to acetic acid, growing well in media containing up to 1.5% (*v*/*v*) acetic acid and 0.5% (*v*/*v*) ethanol, as well as in media with up to 1% acetic acid (*v*/*v*) supplemented with 1% (*v*/*v*) ethanol. Compared to other species within the AAB group, the acetic acid tolerance of Hr-1-5 is relatively low. However, this is consistent with the characteristics of the original isolation source, pear vinegar, which contained 1% (*v*/*v*) ethanol and only 0.54% (*w*/*v*) titratable acids [24]. In addition to the RAE medium with 1% ethanol and 1% acetic acid from which it was originally isolated, Hr-1-5 can also grow on MA, GY, and RAE agar media lacking ethanol and acetic acid. Strain Hr-1-5 was found to be motile, distinguishing it from *G. liquefaciens* and *G. dulcium,* which are non-motile.

Strain Hr-1-5 was capable of growing under microaerobic conditions. However, no growth was observed under strictly anaerobic conditions. Although members of the genus *Gluconacetobacter* are classified as obligate aerobes [59], strain Hr-1-5 demonstrates the ability to grow in environments with reduced oxygen availability. The capacity of certain AAB to grow under low-oxygen conditions has been reported previously [60].

The phenotypic characteristics of strain Hr-1-5 were compared to those of the closest type strains based on dDDH and ANIb values, namely *G. dulcium* LMG 1728^T^, *G. takamatsuzukensis* T61213-20-1a^T^**,** *G. liquefaciens* LMG 1382^T^, *G. asukensis* LMG 27724^T^, *Gluconacetobacter aggeris* LMG 27801^T^, and *G. tumulicola* LMG 27725^T^. Strain Hr-1-5 can be distinguished from the type strains of *G. takamatsuzukensis*, *G. dulcium, G. asukensis*, and *G. tumulicola* by its inability to produce 2-keto-D-gluconic acid. In contrast, Hr-1-5 produces 5-keto-D-gluconic acid, which differentiates it from *G. dulcium* and *G. tumulicola*. Unlike *G. liquefaciens*, Hr-1-5 is capable of growth in a medium where D-ribose, sorbitol, or glycerol serves as the carbon source. It can also grow in media containing D-mannitol, D-gluconate, 1-propanol, or ethanol as a carbon source. None of the tested strains, including Hr-1-5, were able to grow in the presence of 30% D-glucose. Hr-1-5 can utilize ammoniacal nitrogen in Hoyer–Frateur medium supplemented with ethanol, distinguishing it from *G. asukensis* and *G. tumulicola*, which lack this capability. The predominant cellular fatty acid was C_18:1_ *ω*7*c* (cis-vaccenic acid), with 65.4% (Table A1). The high abundance of cis-vaccenic acid is a characteristic feature of AAB [35].

### 3.2. Phylogenetic Analysis

**Figure 3:**
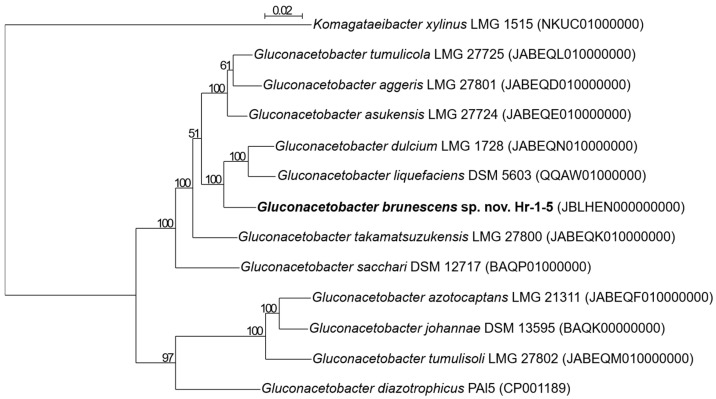
Phylogenetic reconstruction based on core genomes of the type strains within *Gluconacetobacter* clade. The analysis included 73 core genes. The tree was constructed using the maximum-likelihood method, with bootstrap values from 1000 replicates shown at the nodes. The scale bar indicates 0.02 substitutions per site.

Analysis of the 16S rRNA gene revealed the highest identity (98.6%) with *G. diazotrophicus* PAI 5. Based on comparison of the 16S-23S rRNA gene ITS region, strain Hr-1-5 showed the highest sequence identity (92.1%) with *G. liquefaciens* LMG 1382^T^. According to a phylogenetic tree constructed from core genome sequences, the closest relatives of Hr-1-5 are *G. dulcium* LMG 1728^T^, *G. liquefaciens* DSM 5603^T^, and *G. takamarsuzukensis* LMG 27800^T^ (Figure 3). Based on ANIb values (Table S1), strain Hr-1-5 is most closely related to *G. liquefaciens* NBRC 12388^T^ (90.6%), followed by *G. dulcium* LMG 1728^T^ (90.5%), *G-asukensis* LMG 27724^T^ (87.9%), and *G. takamatsuzukensis* LMG 27800^T^ (87.6%). All these values fall below the 95% threshold [61] for species delineation. In silico DNA–DNA hybridization analysis further confirmed that strain Hr-1-5 represents a novel species within the *Gluconacetobacter* genus, showing less than 70% identity to other described species in this genus (Table S2), thereby meeting the accepted criteria for species delineation [61].

### 3.3. Genomic Analysis

The genome size of Hr-1-5 is 2.75 Mbp, with a G+C genomic content of 64.8%. A total of 4182 genes were identified, 4037 code for proteins, while 63 are RNA genes. Among the RNA genes, three complete rRNA operons (5S, 16S, 23S) and 50 tRNA genes were identified. Additionally, 4 non-coding RNAs were identified. A total of 82 pseudogenes were detected, with 39 caused by frameshifts, 57 incomplete, 8 containing internal stop codons, and 20 exhibiting multiple issues.

PHASTEST detected two prophage regions (Table S3): one incomplete (26.1 kb), most similar to *Rhizobium* phage vB RleM PPF1 and *Burkholderia* Bcep176, and one intact (27.1 kb), homologous to *Azospirillum* phage Cd and *Enterococcus* phage phi P27, and containing 28 ORFs, suggesting potential functionality. Cenote-Taker 2 also detected larger prophage regions (Table S4), with the most extensive measuring 60.75 kb and containing a virus hallmark count (represents signature viral genes) of nine, suggesting that a region very likely belongs to a true virus. Another identified region measured 23.2 kb and has a virus hallmark count of three. Previous studies have already described the presence of prophages in AAB. For example, Qian et al., 2022 [47] analyzed 148 *Acetobacter* genomes and identified 350 active prophages, mainly from *Caudovirales* order, which influence genome stability and environmental adaptation. These prophages also carry toxin-antitoxin systems and CRISPR-Cas elements, impacting bacterial defense and gene transfer [62]. The presence of multiple prophage regions in the Hr-1-5 genome, particularly the intact 27.1 kb region, suggests a potential influence on the strain’s genetic plasticity and adaptation to acidic vinegar environments. However, it remains unclear whether these prophages are active or merely cryptic remnants of past infections.

### 3.4. Pigment Synthesis

**Figure 4:**
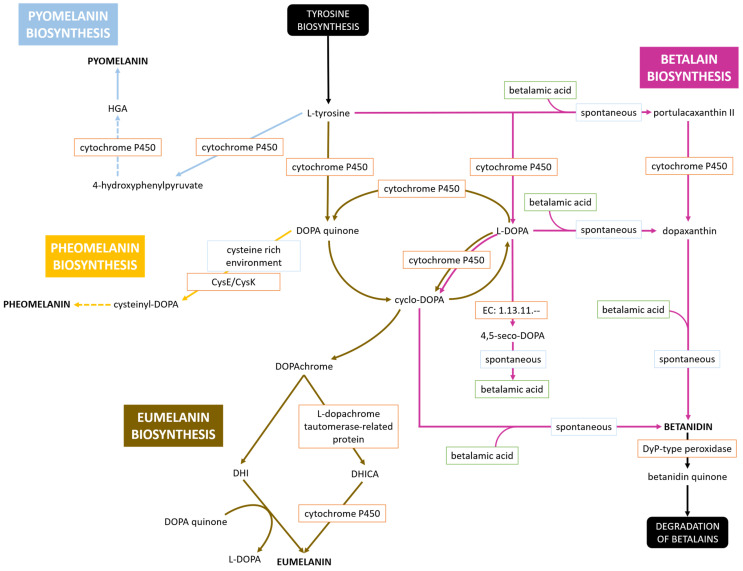
Putative biosynthetic pathways for melanin and betalain in *Gluconacetobacter brunescenes* Hr-1-5. Reactions are indicated by arrows in different colors (light blue for pyomelanin biosynthesis, yellow for pheomelanin biosynthesis, brown for eumelanin biosynthesis, and pink for betalain biosynthesis). Enzymes are shown in orange-bordered boxes. Dashed arrows indicate multiple spontaneous intermediate reactions that are not shown explicitly. In the light blue branch (pyomelanin), cytochrome P450 may also participate in one or more of these reactions.

Hr-1-5 produces a water-soluble brown pigment in RAE medium, as well as in RAE medium supplemented with 1% acetic acid and 1% ethanol, a characteristic not observed in *G. asukensis* and *G. aggeris*. Interestingly, a similar dark coloration was observed in the vinegar from which the strain was originally isolated. After approximately three weeks at room temperature, the vinegar developed a dark brown hue, possibly due to the proliferation of Hr-1-5 and subsequent pigment production in the liquid medium. The production of water-soluble brown pigments has previously been studied in numerous bacteria (see Section 2.5), most of which are associated with L-DOPA. To elucidate the genetic basis of this process in strain Hr-1-5, all L-DOPA-associated pigment biosynthetic pathways currently annotated in the KEGG and Reactome databases were systematically compiled and examined. Four potential outcomes are shown in Figure 4, leading to the production of pyomelanin, pheomelanin, eumelanin, or betanidin. The key branching point is the L-DOPA molecule, from which the pathways diverge toward either melanin or betalain biosynthesis. The core enzyme involved in these processes is likely cytochrome P450, which has been identified as an alternative to monooxygenases such as tyrosinase [63]. Being a substitute for tyrosinase it can lead to synthesis of betalains, specifically betanidin in our case (Figure 4). The betalain biosynthesis pathway was reconstructed using KEGG as a reference [64] and terminates at betanidin, as the genome lacks genes encoding aromatic-L-amino-acid (L-tryptophan) decarboxylase (EC: 4.1.1.28) and cyclo-DOPA 5-O-glucosyltransferase (EC: 2.4.1.-). Under stressful conditions, Dyp-type peroxidase can degrade betanidin to betanidin quinone, which then undergoes further degradation [65]. From L-tyrosine and L-DOPA, the pathways diverge into production of melanin biosynthesis routes. Eumelanin, an insoluble dark brown-black pigment [66], might also be produced in the medium simultaneously, potentially contributing to the observed pigmentation. The eumelanin biosynthesis pathway is very complex (Figure 4), which involves cycling among three key molecules: DOPA quinone, cylco-DOPA, and L-DOPA. Cyclo-DOPA can undergo ring rearrangement to form DOPAchrome, which with the assistance of L-dopachrome tautomerase-related protein and cytochrome P450, is converted into eumelanin [41,42]. In a cysteine-rich environment or in the presence of active cysteine synthases, DOPA quinone can be converted to cysteinyl-DOPA, progressing toward pheomelanin biosynthesis (Figure 4) [41,42]. An alternative pathway may occur before the conversion of L-tyrosine to L-DOPA, leading to the formation of pyomelanin [22] (Figure 4).

**Figure 5:**
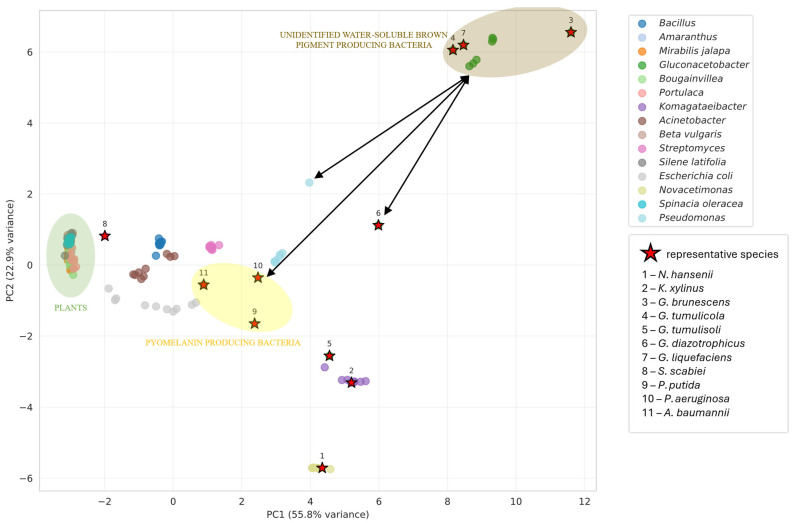
PCA plot for the combined enzyme set P1–P8 of the full dataset. The first two principal components (PC1 and PC2) together account for 78.7% of the total variance (Figure S1). The enzyme profile of Hr-1-5 aligns most closely with pyomelanin from *P. aeruginosa* and betalain of *G. diazotrophicus*. The nearest taxonomic cluster corresponds to the *Pseudomonas* group, whereas plant-derived groups are clearly segregated from bacteria groups. Each colored point in the plot represents an individual bacterial or plant species, with bacterial reference taxa highlighted by red stars and numbered labels to denote representative species.

**Figure A1:**
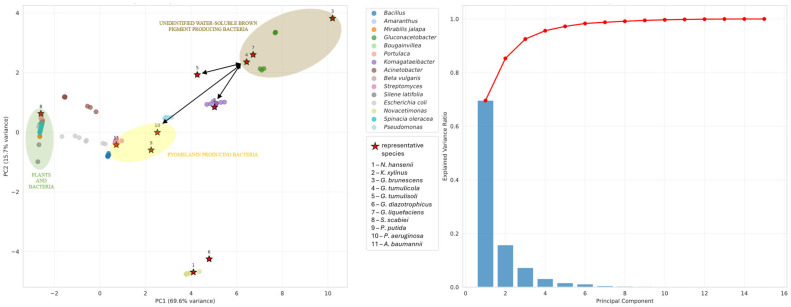
Principal component analysis and the corresponding explained variance plots for the combined enzyme set P1, P2, P4, P5 and P6, representing the intersection of melanin and betalain biosynthesis pathways. The first two principal components (PC1 and PC2) together account for 85.3% of the total variance, indicating strong data structure and high interpretability. Within the PCA space, the enzyme profile of *G. brunescens* Hr-1-5 is most closely associated with eumelanin from *G. tumulisoli*, with the nearest related cluster corresponding to the *Komgataeibacter* group. Among taxa from other genera, pyomelanin-producing *Pseudomonas* species represent the closest characterized pigment pathway. Each colored point denotes an individual bacterial or plant species, while bacterial reference taxa are highlighted with red stars and numeric labels to indicate representative species.

**Figure A2:**
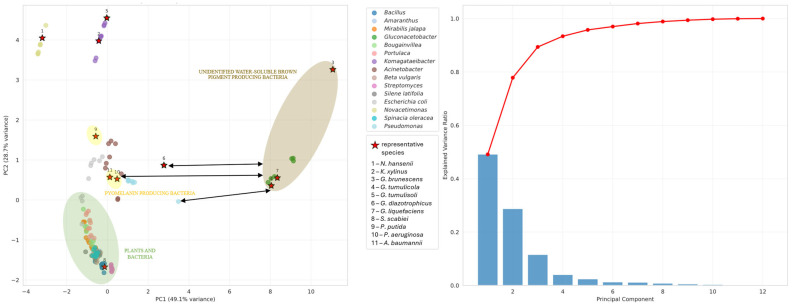
Principal component analysis and associated explained variance plots for the combined enzyme set P1, P2, P7, and P8, corresponding to the betalain biosynthesis. The first two principal components (PC1 and PC2) account for 77.8% of total variance, indicating a well-structured dataset for biological interpretation. Within the ordination space, the enzyme profile of *G. brunescens* Hr-1-5 is positioned closest to pyomelanin-producing *Pseudomonas aeruginosa* and betalain-producing *Gluconacetobacter diazotrophicus*. The nearest related taxonomic cluster corresponds to the *Pseudomonas* lineage. Each colored point represents an individual bacterial or plant species, and bacterial reference taxa are indicated by red stars with numeric labels to denote representative species.

**Figure A3:**
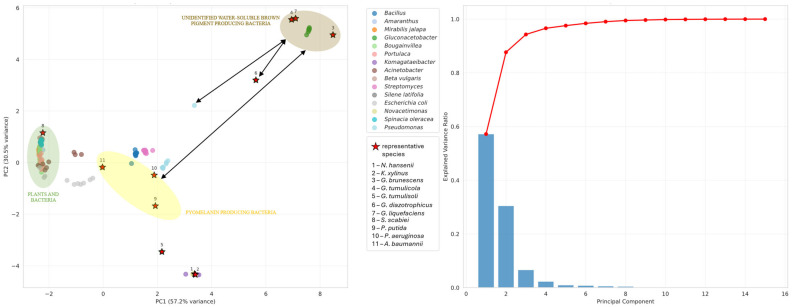
Principal component analysis and the corresponding explained variance plots were generated for the combined enzyme set P4, P5, P6, P7, and P8, associated with the eumelanin biosynthesis pathway. The first two principal components (PC1 and PC2) collectively explain 87.7% of the total variance, indicating a highly structured dataset and strong potential for biological interpretation. Within the PCA ordination, the enzyme profile of *G. brunescens* Hr-1-5 is positioned closest to betalain-producing *Gluconacetobacter diazotrophicus*, whereas the nearest characterized melanin pathway is pyomelanin from the *Pseudomonas* lineage. Each colored point represents an individual bacterial or plant species, with bacterial reference taxa highlighted by red stars and numeric labels to indicate representative species.

To determine the most probable pigment biosynthetic pathway in *G. brunescens* Hr-1-5, we performed a multivariate statistical analysis of enzyme-associated features using a comparative dataset comprising 151 species and 24 variables. The variables include eight measures of query coverage, eight of percent identity, and eight of log-transformed E-value. Principal component analysis (PCA) of the enzyme dataset produced PCA plots (Figure 5, Figure A1, Figure A2 and Figure A3). In each case, the first two principal components accounted for >70% of the total variance, indicating a well-structured dataset amenable to biological interpretation. Data points marked with starts corresponding to the taxonomic groups described in Section 2.5. Across all PCA plots, strain Hr-1-5 clusters consistently with *G. liquefaciens*, *G. tumulicola*, as well as *G. dulcium*, of which pigment production is not studied. All of them, with the exception of the latter, produce an unidentified water-soluble brown pigment. Analysis of the complete dataset suggested betalain as the closest pigment type overall; however, optimal clustering of the Fr-1-5 enzyme profiles supported pyomelanin as the most likely pigment candidate (Figure 5).

**Figure 6:**
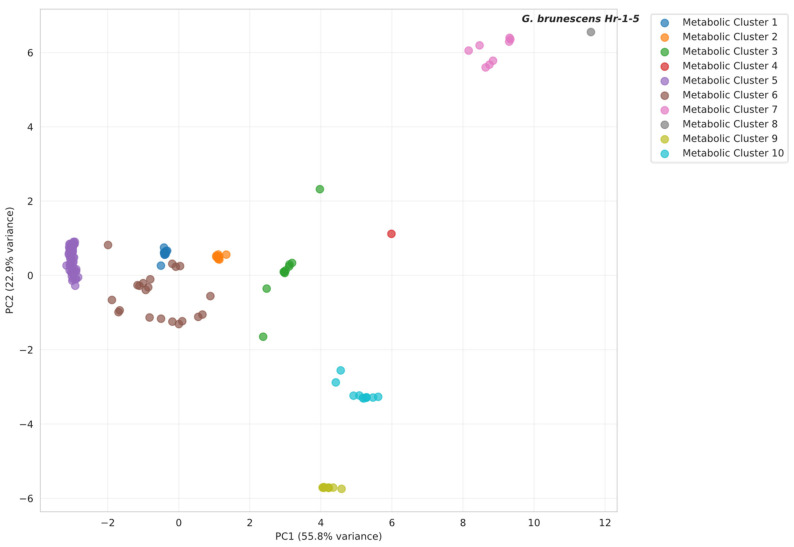
Hierarchical clustering analysis, where *G. brunescens* Hr-1-5 is clearly delineated and assigned to a distinct metabolic cluster marked with a gray dot.

The subset analyses corroborate the interpretation derived from the preceding PCA, supporting melanin biosynthesis as the most plausible pathway. At the intersection of the betalain and melanin networks (Figure A1), the trajectory of the enzymes profiles indicates a shift toward melanin production. This is evident by the pronounced separation of *G. diazotrophicus* and plant taxa from strain Hr-1-5, whereas pyomelanin-producing bacteria and the eumelanin-producing bacterium exhibit the greatest proximity in ordination space. In contrast, analyses restricted to the betalain pathway (Figure A2) or the eumelanin pathway (Figure A3) did not yield statistically significant patterns that would further clarify the relationship of the unidentified water-soluble pigment to any characterized pigment class. To refine these findings, we subsequently performed metabolic clustering on the complete dataset, which notably represented the only analysis in which plant and bacterial taxa remained entirely segregated. To determine the optimal cluster number, two complementary approaches were employed: the elbow method, which indicated six clusters, and the silhouette analysis, which favored ten clusters (Figure S2). On the basis of these criteria and to capture the finer structure suggested by the silhouette score, the final clustering solution was set at ten clusters. Following selection of the optimal cluster number, hierarchical clustering was performed (Figure 6). Application of a cut-off line corresponding to ten clusters confirmed the suitability of this partitioning, as evidenced by the substantial Euclidean distances separating the resulting groups (Figure S3). Based on this analysis, *G. brunescens* Hr-1-5 was placed in metabolic cluster 8, which contains no other taxa. The nearest neighboring cluster is cluster 7, comprising *G. dulcium*, *G. liquefaciens*, and *G. tumulicola*. Among clusters with characterized pigments, cluster 4 representing betalain biosynthesis is the closest, followed by cluster 3, which contains the *Pseudomonas* group associated with pyomelanin biosynthesis. Despite its slightly greater Euclidean distance, cluster 3 exhibits a higher mean silhouette coefficient, indicating a more cohesive and statistically robust grouping than cluster 4. Interestingly, cluster 4 consists solely of *G. diazotrophicus* reflecting its unique metabolic profile, while plant species segregate exclusively into cluster 5, underscoring the clear metabolic divergence between plant and bacterial taxa.

A comprehensive silhouette analysis and K-means clustering with assigned centroids were further conducted (Figure S4). The silhouette plot indicates which metabolic clusters exceed the average silhouette score threshold of 0.680. Notably, metabolic cluster 8, containing *G. brunescens* Hr-1-5, falls below this threshold, suggesting that the strain could alternatively be associated with cluster 3. K-means clustering assignments correspond closely to the hierarchical clustering results, providing visual confirmation of the optimal cluster number. Based on these findings, we hypothesize that the unidentified water-soluble brown pigment produced by *G. brunescens*, *G. liquefaciens* and *G. tumulicola* is closely related to pyomelanin, consistent with observed similarities in solubility, thermal and photostability, and antimicrobial effects. However, notable difference exists between the metabolic clusters of these bacteria and the pyomelanin-producing *Pseudomonas* group. For instance, *Pseudomonas* species possess the 4-hydroxyphenylpyruvate dioxygenase (HPPD) gene, which product catalyzes conversion of 4-hydroxyphenylpyruvate to homogentisic acid (HGA), whereas members of the *Gluconacetobacter* group lack this enzyme. Some organisms utilize cytochrome P450 enzyme as a functional substitute for HPPD via alternative homogentisate pathways. Example include OhpA (CYP116B) in *Cupriavidus pinatubonensis*, converting 2-hydroxyphenylacetate to homogentisate, and PhacB (CYP504B) [67] in *Aspergillus nidulans*, hydroxylating 3-hydroxyphenylacetate to homogentisate [68]. Comparative analysis of cytochromes from Hr-1-5 against these enzymes yielded less than 40% sequence identity and E-value exceeding 1 × 10^−40^, indicating low similarity and no statistically significant homology. From these findings, several conclusions can be drawn:

(a) The unidentified water-soluble brown pigment of *G. brunescens*, *G. liquefaciens* and *G. tumulicola* is a novel pigment of the *Gluconacetobacter* group;
(b) The unidentified water-soluble brown pigment is pyomelanin, but the cytochromes P450 of these bacteria may follow a novel, yet undescribed pathway;
(c) *G. dulcium* requires thorough investigation to determine whether it also produces the water-soluble brown pigment;
(d) All *Gluconacetobacter* species warrant further study to elucidate their pigment biosynthesis pathways.

### 3.5. Bacterial Nanocellulose Synthesis and Co-Cultivation

Strain Hr-1-5 did not produce bacterial nanocellulose. To further investigate its potential for cellulose biosynthesis, we analyzed its genome for the presence of *bcs*1, *bcs*2, *bcs*3, and *bcs*4 operons [69]. Of these, only the *bcs*2 operon was identified. The absence of the *bcs*1 in the strain Hr-1-5 suggests a loss of cellulose synthesis capability.

**Figure 7:**
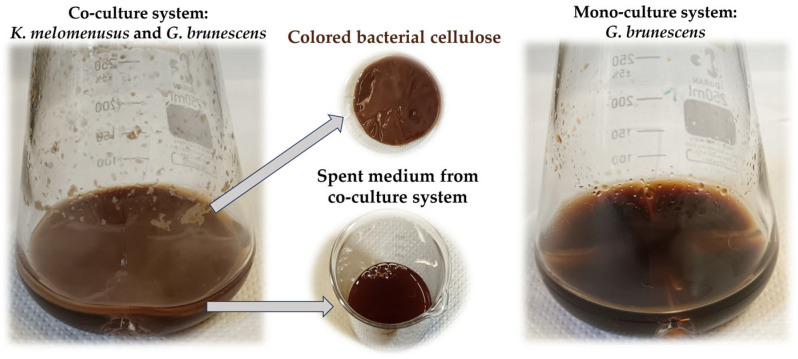
The pictures show the growth of *G. brunescens* in co-culture with *K. melomenusus* and in monoculture in RAE medium containing 1% ethanol and 1% acetic acid. The diameter of the colored bacterial cellulose is 80 mm.

Additionally, we performed a co-cultivation experiment using the bacterial cellulose-producing strain *Komagataeibacter melomenusus* AV436 in combination with the pigment producing strain, *Gluconacetobacter brunescens* Hr-1-5. The co-culture produced dark brown bacterial cellulose (Figure 7). This experiment demonstrates that it is feasible to combine different bacterial species under the same conditions to naturally produce colored bacterial cellulose.

### 3.6. Antimicrobial Resistance and Inhibitory Effects

Strain Hr-1-5 exhibited resistance to gentamicin, ampicillin, ciprofloxacin, erythromycin, trimethoprim, and chloramphenicol. Furthermore, it demonstrated growth-inhibitory effects against *E. coli* ATCC 25922, *E. faecalis* ATCC 29212, and *S. aureus* ATCC 29213, while *K. melomenusus* AV436^T^ remained unaffected. This inhibition may result from the antimicrobial properties of the pigments produced by Hr-1-5, which could be active at high concentrations. Both betalains and melanins are known to possess antimicrobial activity, active also against antibiotic-resistant bacteria [70,71]. This suggests that if the pigments synthesized by Hr-1-5 are indeed melanins, they may account for the observed inhibitory effects. In contrast, *K. melomenusus* may exhibit resistance, possibly due to its adaptation to the vinegar environment.

## 4. Description of *Gluconacetobacter brunescens* sp. nov.

*Gluconacetobacter brunescens* (bru.nes’ cens. L.adj. brunescens, N.L. masc. part. adj. *brunescens*, becoming brown, referring to the characteristic brownish coloration).

Cells are Gram-stain-negative, approximately 3.3 µm in length and 0.5 µm in width, and motile. Colonies grown on RAE medium supplemented with 1% acetic acid and 1% ethanol at 30 °C for 3 days are brown, round, raised, with smooth edges, and measure approximately 0.4 mm in diameter. The strain is oxidase-negative and catalase-positive. It grows on RAE, MA, GY media, and RAE supplemented with 1% acetic acid and 1% ethanol. The strain oxidizes ethanol to acetic acid and can grow under both aerobic and microaerobic conditions. No growth is observed in the presence of 10% ethanol, while weak growth occurs with 1% NaCl. No growth is detected with 2% NaCl or 30% D-glucose. The strain is capable of utilizing D-ribose, sorbitol, D-mannitol, D-gluconate, glycerol, 1-propanol, and ethanol as carbon sources. It produces 5-keto-D-gluconic acid from D-glucose but does not produce 2-keto-D-gluconic acid. The strain tolerates up to 1.5% acetic acid in the presence of 0.5% ethanol and up to 1% acetic acid with 1% ethanol. It can utilize (NH_4_)_2_SO_4_ as the sole nitrogen source in the Hoyer–Frateur and Asai medium with D-mannitol, and in Hoyer–Frateur medium with ethanol. A weak ability to utilize (NH_4_)_2_SO_4_ as the sole nitrogen source is observed in Hoyer–Frateur medium with glucose and Asai medium with ethanol, whereas it is unable to use (NH_4_)_2_SO_4_ as the sole nitrogen source in the Hoyer–Frateur with glucose.

The type strain Hr-1-5^T^ (=ZIM B1168^T^ = LMG 33629^T^) was isolated from pear vinegar in Pohorje, Slovenia.

The 16S rRNA gene and whole-genome sequences of strain Hr-1-5^T^ have been deposited at DDJB/ENA/GenBank under the accession numbers PV083164 and JBLHEN000000000, respectively. The 16S-23S rRNA gene ITS region is deposited under accession number PV083165.

## Data Availability

The original contributions presented in the study are included in the article and Supplementary Materials, further inquiries can be directed to the corresponding author. The source code supporting this study is available at GitHub (v3.18.1).

## Supplementary Materials

The following supporting information can be downloaded at: https://www.mdpi.com/article/10.3390/microorganisms13112620/s1, Table S1: ANIb analysis of draft genome Hr-1-5 compared to other *Gluconacetobacter* type strains. Table S2: In silico DNA–DNA hybridization (dDDH) analysis of the draft genome Hr-1-5 compared to other *Gluconacetobacter* type strains. Table S3: Prophage identification in Hr-1-5 genome using PHASTEST. Table S4: Prophage identification in the Hr-1-5 genome using Cenote-Taker 2. Figure S1: PCA explained variance corresponding to the analysis shown in Figure 5. The combined explained variance ratio of PC1 and PC2 already accounts for a substantial 78.7% of the total variance. Figure S2: The elbow method plot (left) and silhouette analysis plot (right) with the dashed lines indicating the optimal number of clusters identified by each method. Figure S3: A hierarchical clustering dendrogram with the cut-off line shows ten clusters. Figure S4: The silhouette plot (left) and K-means plot (right) show the clustering results with the dashed line marking the highest average silhouette score. *G. brunescens* remained in the same metabolic cluster even after K-means clustering method.

## Appendix A

**Table A1:** Cellular fatty acid profiles of strain Hr-1-5.

Fatty Acid (%)	Hr-1-5 (%)
C_12:0_	0.3
C_14:0_	3.5
C_16:0_	12.6
C_17:0_	0.2
C_18:0_	1.9
C_16:1_ ω7c/C_16:1_ ω6c	0.7
C_18:1_ ω7c	65.4
C_14:0_ 2OH	4.9
C_16:0_ 2OH	5.8
C_16:0_ 3OH	1.4
C_18:0_ 3OH	0.5
C_14:0_ 3OH/C_16:1_ iso I	0.9
C_19:0_ cyclo ω8c	1.1
