# Supplementary material for: Gluconacetobacter brunescens sp. nov., a Novel Acetic Acid Bacterium Isolated from Pear Vinegar, Producing a Water-Soluble Brown Pigment
Source: Microorganisms. 2025 Nov 18;13(11):2620. doi: 10.3390/microorganisms13112620 (PMC12654741; doi:10.3390/microorganisms13112620)
Supplement: Supplementary file 1 [file microorganisms-13-02620-s001.zip › microorganisms-3877111-supplementary.pdf]

Table S1. ANIb analysis of draft genome Hr-1-5 compared to other *Gluconacetobacter* type strain.

	ANIb Hr-1-5 (%)
Gluconacetobacter liquefaciens NBRC 12388^T	90.56
Gluconacetobacter dulcium LMG 1728^T	90.52
Gluconacetobacter asukensis LMG 27724^T	87.94
Gluconacetobacter takamatsuzukensis LMG 27800^T	87.61
Gluconacetobacter aggers LMG 27801^T	87.58
Gluconacetobacter tumulicola LMG 27725^T	87.53
Gluconacetobacter sacchari DSM 12717^T	85.74
Gluconacetobacter diazotrophicus PA1 5^T	79.89
Gluconacetobacter johannae DSM 13595^T	78.51
Gluconacetobacter tumulisoli LMG 27801^T	78.36
Gluconacetobacter azotocaptans DSM 13594^T	78.15

Table S2. *In-silico* DNA-DNA hybridization (dDDH) analysis of draft genome Hr-1-5 compared to other *Gluconacetobacter* type strains.

	Hr-1-5 (%)
Gluconacetobacter dulcium LMG 1728	46.0
Gluconacetobacter liquefaciens DSM 5603	45.8
Gluconacetobacter asukensis LMG 27724	37.6
Gluconacetobacter tumulicola LMG 27725	37.3
Gluconacetobacter takamatsuzukensis LMG 27800	37.2
Gluconacetobacter aggeris LMG 27801	37.0
Gluconacetobacter sacchari DSM 12717	33.2
Gluconacetobacter diazotrophicus PAI 5	24.9
Gluconacetobacter johannae DSM 13595	23.5

Table S3. Prophage identification in Hr-1-5 genome using PHASTEST.

Bacterial Host Strain	Region Length (kb)	Completeness	The Number of ORFs present in the region	Region Position	Two Most Common Phage Species
Hr-1-5	26.1	incomplete	30	185704-211886	*Rhizobium* phage vB RleM PPF1<br>*Burkholderia* Bcep176
Hr-1-5	27.1	intact	28	238622-266045	*Azospirillum* phage Cd<br>*Enterococcus* phage phiP27

Table S4. Prophage identification in the Hr-1-5 genome using Cenote-Taker 2.

Bacterial Host Strain	Region Length (kb)	Region Position	Hallmark Count
Hr-1-5	60.75	2391300- 2452050	9
Hr-1-5	23.20	2342250- 2365450	3

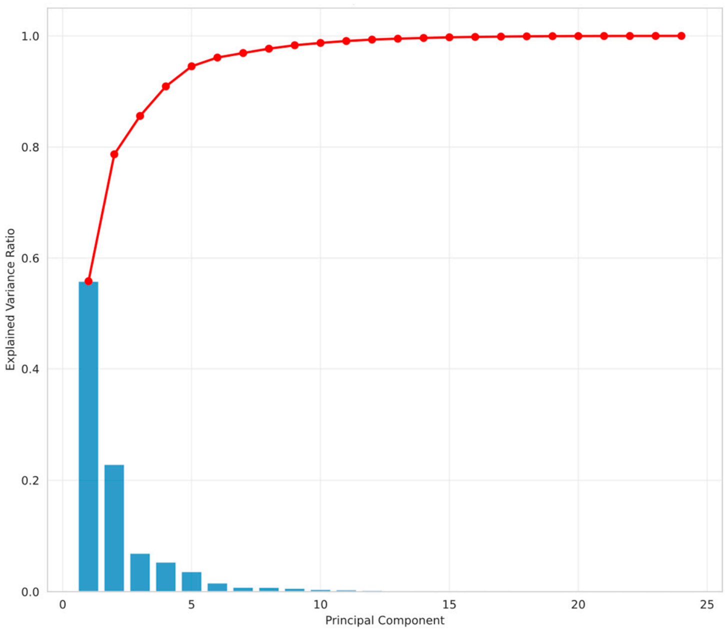

Figure S1. PCA explained variance corresponding to the analysis shown in Figure 5. The combined explained variance ratio of PC1 and PC2 already accounts for a substantial 78.7% of the total variance.

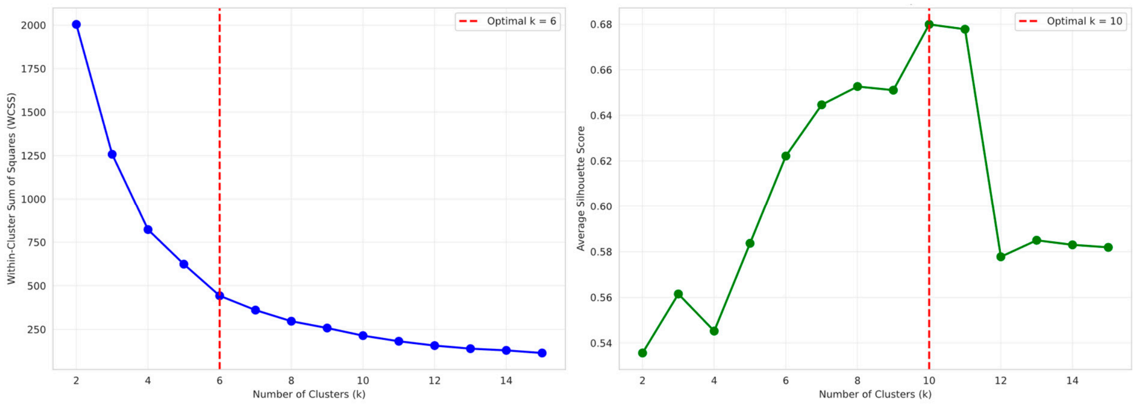

Figure S2. The elbow method plot (left) and silhouette analysis plot (right) with the dashed lines indicating the optimal number of clusters identified by each method.

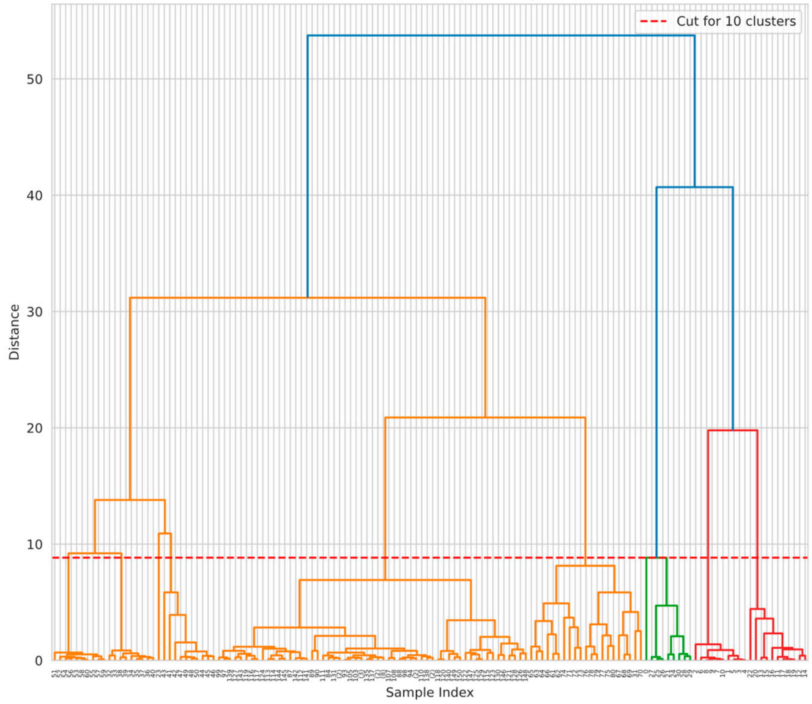

Figure S3. A hierarchical clustering dendrogram with marked cut-off line shows ten clusters.

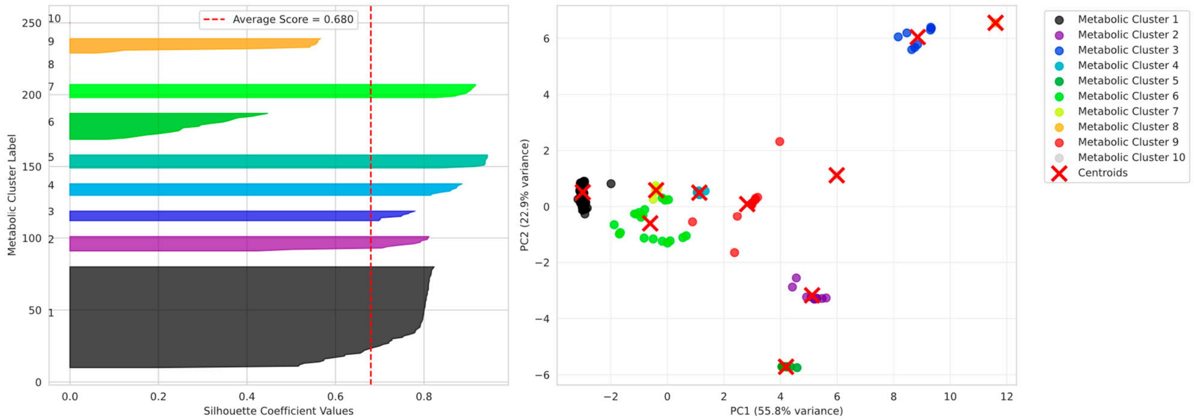

Figure S4. The silhouette plot (left) and K-means plot (right) show the clustering results with the dashed line marking the highest average silhouette score. *G. brunescens* remained in the same metabolic cluster even after K-means clustering method.
